# Characteristics of Brazilian school food and physical activity environments: PeNSE 2015

**DOI:** 10.11606/s1518-8787.2021055003377

**Published:** 2021-12-16

**Authors:** Lucyane Barbosa Oliveira Souza, Ana Beatriz Coelho de Azevedo, Daniel Henrique Bandoni, Daniela Silva Canella

**Affiliations:** I Universidade do Estado do Rio de Janeiro Programa de Pós-Graduação em Alimentação, Nutrição e Saúde Rio de Janeiro RJ Brasil Universidade do Estado do Rio de Janeiro. Programa de Pós-Graduação em Alimentação, Nutrição e Saúde. Rio de Janeiro, RJ, Brasil; II Universidade Federal de São Paulo Instituto de Saúde e Sociedade Departamento Saúde, Clínica e Instituições Santos SP Brasil Universidade Federal de São Paulo. Instituto de Saúde e Sociedade. Departamento Saúde, Clínica e Instituições. Santos, SP, Brasil; III Universidade do Estado do Rio de Janeiro Instituto de Nutrição Departamento de Nutrição Aplicada Rio de Janeiro RJ Brasil Universidade do Estado do Rio de Janeiro. Instituto de Nutrição. Departamento de Nutrição Aplicada. Rio de Janeiro, RJ, Brasil

**Keywords:** School Feeding, Exercise, Education, Primary and Secondary, School Health Services, Health Promotion

## Abstract

**OBJECTIVE:**

To characterize the food and physical activity environments in Brazilian public and private schools, and develop indicators to evaluate them.

**METHODS:**

This is a cross-sectional study conducted with data from a questionnaire on school characteristics of the 2015 National Adolescent School-based Health Survey, answered by principals or coordinators, referring to 3040 public and private schools throughout the country. The variables related to food and physical activity environments were described in isolation, and an indicator was developed for each environment, with scores ranging from 0 to 100. The frequency and mean score of each variable were described according to the administrative sphere (public or private).

**RESULTS:**

The public sector showed a predominance of school meals offer (97.8%), whereas the private sector, of canteens (89.8%). Both had a similar frequency of alternative food outlets in the surroundings. Private schools provided all markers of healthy and unhealthy eating in canteens more frequently. Public schools scored higher in “Food and beverage availability” (64.9) than private schools (55.8). The characteristics of physical activity environments showed that sports courts and sports or games equipment were common in public (69.2% and 90.7%, respectively) and private schools (94.1% and 99.8%, respectively), though at a significantly higher frequency in the second group. Private schools scored higher in “Structures and materials availability” than public schools (63.3 and 41.6, respectively).

**CONCLUSIONS:**

Public schools provide a more favorable food environment, whereas private schools, a physical activity environment.

## INTRODUCTION

Non-communicable diseases are one of the major public health issues in Brazil and the world whose main risk factors are unhealthy eating and physical inactivity^1^. Behaviors acquired in childhood and adolescence tend to perpetuate themselves in adulthood impacting adults’ quality of life, thus making health promotion essential at this stage^2^.

Schools are spaces for socializing and interacting. Thus, international organizations^3^ and Brazilian public policies^4^ recognize it as a strategic place for health-promoting actions. Implementing these actions can contribute to an adequate and healthy diet, and regular physical activity. In Brazil, the *Programa Nacional de Alimentação Escolar* (Brazilian school feeding program - PNAE) guarantees a universal meal offer for public schools^5^, which may have canteens (regulated or not by local governments^7,8^) and alternative food outlets around them^8^. Private schools lie outside the PNAE, but may offer meals, have canteens, alternative food outlets, and vending machines^8^. Physical education is part of school curricula^9^, and alongside existing structures/facilities and extracurricular school activities^10,11^, constitute a window of opportunity for students to adhere to physical activities.

Evidence shows that the school environment influences students’ nutrition^12^ and physical activities^10,16,17^, assessing it mainly via the availability of food and beverages, and structures and activities, respectively. They also indicate important differences between public and private schools, and among Brazilian regions^8,18^. The National Adolescent School-based Health Survey (PeNSE) evaluates aspects of school food and physical activity environments. A study with data representative of the country exploring these aspects, the indicators constructed from these variables, and their geographical differences can contribute to the knowledge and monitoring of school environments, and the formulation of health-promoting actions.

Therefore, this study aimed to characterize the food and physical activity environments of Brazilian public and private schools, and develop indicators to evaluate them.

## METHODS

This is a cross-sectional study using data from PeNSE 2015, conducted by the Brazilian Institute of Geography and Statistics (IBGE) with the Ministries of Health and Education.

Data from Sample 1 of PeNSE 2015, including 3040 public and private schools with 9th grade elementary classes, distributed in 675 Brazilian municipalities were used. The sample was sized to estimate population parameters (prevalence or proportion) in different Brazilian geographic domains: 26 capitals and the Federal District, 26 Federation Units (covering municipalities other than the capitals), five macro-regions, and Brazil, totaling 53 geographical strata.

The schools in each stratum were selected from the 2013 School Census, and probability of selection was proportional to the number of 9th grade classes. Data collection was performed using an electronic questionnaire, following the Global School-Based Student Health Survey methodology developed by the World Health Organization (WHO), and the modules to assess the environments (the subject of this study) were applied only to school principals or coordinators^19^.

To characterize school food environments, the following variables were considered: presence of usable kitchens; usable cafeterias; vegetable gardens; school meals offered; available drinking water; presence of canteens; of alternative food outlets at the school entrance or surroundings. In the case of canteens or alternative food outlets, the following variables were evaluated: 1) Natural fruit juices or refreshments; 2) fresh fruit or fruit salads; 3) sweetened beverages (including soft drinks); 4) packaged salty snacks; 5) savory or sweet crackers; and 6) candy and others.

Physical activity environments were evaluated by the presence of: sports courts; at least one indoor sports court; running/athletics tracks; halls; swimming pools; changing rooms; sports or games equipment.

A dialogue between this study and theoretical-conceptual models of food and physical activity environments are allowed by the variables available in the PeNSE questionnaire. Considering schools as organizational food environments, institutional sphere (kitchens, cafeterias, vegetable gardens, canteens, and alternative food outlets) and the dimension of availability in the establishments’ sphere are expressed by the available PeNSE variables^20^. Avaliable built external environments are represented by physical activity variables^21^.

In view of these dimensions, three indicators were created: two food environment scores (“Availability of production structure and offer of food and beverage” and “Food and beverage availability”), and one physical activity environment score (“Structures and materials availability”). For this, all variables were dichotomized into 1 and 0; 1 for desirable items (e.g., offered school meals, lack of sweetened beverages in canteens, and presence of halls), and 0 for undesirable items (e.g., offer of sweetened beverages in canteens, and lack of sports equipment). In schools lacking a canteen, unhealthy item availability was scored 1, and healthy item availability, 0, due to their absence.

In total, five items were included in the Availability of production structure and offer of food and beverage score: presence of kitchens, cafeterias, vegetable gardens, canteens, and alternative food outlets; in the Food and beverage availability score, 14: school food meals offer; available drinking water; in the case of canteens and alternative food outlets, the offer of: 1) natural fruit juices or refreshments, 2) fresh fruit or fruit salads, 3) sweetened beverages (including sodas), 4) industrialized packaged salty snacks, 5) savory or sweet crackers, and 6) candies, and others; and in Structures and materials availability score, seven: sports courts; at least one indoor sports court; running/athletics tracks; halls; swimming pools; changing rooms; and sports or games equipment. Score range was defined thus: 0 to 5, 0 to 14, and 0 to 7, respectively. Then, scores were standardized on a 0-100-point scale. The internal consistency of each score was evaluated by Cronbach’s alpha coefficient whose value was considered acceptable if alpha ≥ 0.7^22,23^, and item correlation by the Pearson correlation coefficient.

Moreover, geographic variables, such as macro-regions, school location (in capitals or not), and administrative dependence (public or private) were used in the analyses.

Descriptive analyses were conducted via frequency and the 95% confidence interval (95%CI) of each variable. Analyses were carried out for Brazil as a whole, the five macro-regions, and school location, stratified by administrative dependence. Mean score values and 95%CI were estimated according to geographic variables and administrative dependence. The absence of overlap between intervals was considered a significant difference.

The survey module of the statistical software Stata SE version 15.1 (Stata Corp., College Station, USA) was used to estimate the effects of the complex sampling plan of this study.

PeNSE was approved by the National Research Ethics Committee of the Brazilian National Health Council under opinion no. 1006467, on March 30, 2015.

## RESULTS

We analyzed data on the food environment of 2,947 Brazilian schools, and on the physical activity environment of 3,034, of which about 80% were public schools, and 20%, private. The characterization of school food environments by administrative dependence showed the higher prevalence of the school meals offer, and the presence of usable kitchens, and alternative food outlets in public schools, (97.8%, 96.7%, and 30.4% versus 26.2%, 74.9%, and 25.2%, in private schools, respectively), and of canteens in private ones (89.8% versus 33%). More than 98% of schools had available drinking water. (Table 1).

**Table 1:** Characterization of the school food environment for all Brazilian schools according to location and administrative dependence. Brazil, 2015.

Variables	Brazil	Administrative dependence		Location			

				Capital		Other cities	
		
		Public	Private	Public	Private	Public	Private
						
	% (95%CI)	% (95%CI)	% (95%CI)	% (95%CI)	% (95%CI)	% (95%CI)	% (95%CI)
Structures to produce and offer food and beverages							
Usable kitchen	93.6 (92.3–94.7)	96.7 (95.6–97.6)	74.9 (69.0–80.0)	98.2 (97.3–98.8)	73.1 (64.8–80.1)	96.4 (95.0–97.4)	76.2 (67.7–83.0)
Usable mess hall	60.8 (57.8–63.6)	61.0 (57.6–64.2)	59.6 (52.9–66.0)	71.0 (65.6–75.8)	62.9 (54.8–70.4)	58.5 (54.6–62.3)	57.1 (48.2–65.6)
Vegetable garden	27.6 (24.5–30.9)	27.4 (24.0–31.2)	28.6 (22.0–36.2)	22.7 (18.0–28.3)	22.0 (15.1–30.9)	28.6 (24.5–33.0)	33.6 (23.7–45.2)
Canteen and alternative food outlet	11.4 (9.43–13.6)	9.4 (7.4–11.9)	22.9 (20.1–27.5)	12.2 (8.6–17.0)	33.4 (25.3–42.7)	8.7 (6.5–11.7)	15.0 (8.9–24.1)
Canteen only	29.8 (26.6–33.3)	23.6 (20.1–27.5)	66.8 (58.9–73.9)	22.9 (18.0–28.8)	65.2 (56.0–73.3)	23.7 (19.7–28.3)	68.1 (55.3–78.7)
Alternative food outlet only	18.3 (16.3–20.5)	21.0 (18.7–23.5)	2.3 (0.4–11.9)	19.7 (15.3–25.1)	0.0 (0.0–0.2)	21.3 (18.8–24.0)	3.7 (0.1–21.1)
Without canteen and alternative food outlet	40.5 (37.1–44.0)	46.0 (42.2–49.9)	8.0 (3.9–15.5)	45.2 (39.2–51.4)	0.1 (0.0–0.3)	46.2 (41.7–50.9)	13.3 (6.3–25.6)
Food and beverage availability							
School meals	87.5 (85.5–89.3)	97.8 (96.4–98.7)	26.2 (19.7–34.0)	98.2 (97.0–99.0)	25.1 (18.2–33.6)	97.7 (95.9–98.8)	27.0 (17.5–39.1)
Drinking water	98.3 (97.6–98.8)	98.0 (97.2–98.6)	99.9 (99.4–100.0)	99.4 (99.0–99.7)	100.0	97.7 (96.7–98.4)	99.8 (98.9–100.0)
Canteen food and beverage availability (considering only schools with canteens)							
Natural fruit juice/refreshments	78.5 (72.9–83.2)	72.2 (63.8–79.2)	90.2 (86.6–92.9)	77.8 (69.3–84.4)	91.2 (86.6–94.3)	70.5 (60.0–79.2)	89.4 (83.9–93.1)
Fresh fruit or fruit salad	22.1 (18.4–26.4)	8.5 (5.5–12.7)	47.4 (40.3–54.6)	16.9 (9.2–29.2)	56.5 (48.1–64.5)	6.0 (3.4–10.4)	39.3 (28.4–51.3)
Sweetened beverages (including soft drinks)	61.4 (55.7–66.9)	53.3 (45.1–61.2)	76.5 (69.5–82.3)	73.1 (62.4–81.7)	81.1 (76.3–85.0)	47.6 (38.1–57.2)	72.4 (60.3–81.9)
Packaged salty snacks	51.5 (45.6–57.4)	50.5 (42.1–58.8)	53.5 (46.4–60.5)	61.9 (51.3–71.5)	58.3 (49.9–66.3)	47.2 (37.2–57.4)	49.2 (38.6–55.9)
Sweet and savory crackers	43.3 (37.3–49.5)	35.5 (27.7–44.2)	57.6 (50.4–64.4)	40.6 (29.5–52.8)	60.1 (51.4–68.2)	34.1 (24.7–44.8)	55.3 (44.3–65.9)
Candies and others	41.4 (35.8–47.2)	33.1 (25.8–41.3)	56.6 (49.7–63.3)	43.5 (31.7–56.1)	58.8 (50.7–66.5)	30.1 (21.7–40.1)	54.6 (44.2–64.7)
Alternative food outlet food and beverage availability (considering only schools with alternative food outlets)							
Natural fruit juice/refreshment	43.8 (38.2–49.5)	44.1 (38.3–50.1)	41.5 (26.8–57.9)	43.1 (32.6–54.2)	33.8 (18.6–53.3)	44.3 (37.5–51.4)	51.9 (23.4–79.1)
Fresh fruits or fruit salad	7.4 (5.1–10.8)	7.1 (4.6–10.8)	10.2 (4.9–20.2)	4.6 (2.8–7.4)	13.8 (6.1–28.3)	7.7 (4.7–12.4)	5.5 (1.3–20.1)
Sweetened beverages (includes soft drinks)	73.8 (69.2–77.8)	74.6 (69.7–78.9)	68.0 (52.6–80.2)	83.1 (74.6–89.2)	58.6 (39.1–75.7)	72.4 (66.8–77.4)	80.7 (57.3–92.9)
Packaged salty snacks	69.7 (65.1–74.0)	71.4 (66.4–75.9)	57.7 (42.2–71.8)	78.1 (69.3–84.8)	56.5 (37.3–73.9)	69.7 (63.9–74.9)	59.2 (33.2–81.0)
Sweet and savory crackers	39.2 (33.9–44.8)	38.2 (32.5–44.2)	46.4 (30.6–62.9)	40.0 (29.2–51.9)	42.6 (24.7–62.7)	37.7 (31.2–44.7)	51.5 (24.8–77.4)
Candies and others	61.0 (55.5–66.3)	58.9 (52.9–64.7)	76.2 (64.1–85.1)	54.8 (43.2–65.8)	80.7 (70.3–88.0)	60.0 (52.8–66.7)	70.1 (43.1–87.9)

Private schools showed a more frequent availability of fresh fruit and fruit salads in their canteens (47.4%) than public ones (8.5%). Alternative food outlets seldom sold such items, with no difference between administrative dependences. The canteens of both private and public schools mostly had available natural fruit juice/refreshments and sweetened beverages (90.2% and 72.2%, respectively). Alternative food outlets in public schools sold sweetened beverages (74.6%) and packaged salty snacks (71.4%) the most, whereas private schools, candies, and others (76.2%), sweetened beverages (68%), and packaged salty snacks (57.7%). Public school canteens in capitals showed a significantly higher availability of sweetened beverages than in other cities (73.1% versus 47.6%) (Table 1).

We observed homogeneity in the high offer of school meals and presence of usable kitchens in public schools of all Brazilian regions. The Southeast showed the highest (82.2% of public and 71.9% of private schools), whereas the Northeast, the lowest frequency (32.4% of public and 43.6% of private schools) of usable cafeterias in both administrative dependencies, respectively. The Northeast showed the lowest presence of canteens in the public sector (14.4%), but the highest availability of fresh fruit and fruit salads in the existing canteens (16.4%). Of importance is the lack of fresh fruit and fruit salads in alternative food outlets near Southeastern public schools, and its 1.3% insignificant frequency in the private sector. The North and the Midwest showed the greatest difference in the presence of vegetable gardens between public (36.4% and 36.2%) and private schools (9.9% and 23.4%), and the Northeast, the lowest frequency among public schools (20.3%) (Table 2).

**Table 2:** Characterization of the school food environment according to region and administrative dependence. Brasil, 2015.

Variáveis	Region									

	North		Northeast		Southeast		South		Midwest	
				
	Public	Private	Public	Private	Public	Private	Public	Private	Public	Private
									
	% 95%CI	% 95%CI	% 95%CI	% 95%CI	% 95%CI	% 95%CI	% 95%CI	% 95%CI	% 95%CI	% 95%CI
Structures to produce and offer food and beverages										
Usable kitchen	95.1 (91.0–97.4)	74.2 (57.8–85.8)	94.5 (92.0–96.3)	63.5 (54.5–71.7)	99.3 (96.9–99.8)	80.7 (69.8–88.3)	94.7 (89.5–97.5)	86.3 (71.3–94.1)	95.8 (92.7–97.7)	69.2 (56.5–79.5)
Usable mess hall	61.0 (54.5–67.2)	52.3 (36.2–67.8)	32.4 (28.2–36.9)	43.6 (35.7–51.9)	82.2 (75.0–87.7)	71.9 (60.8–80.9)	68.1 (60.4–74.9)	58.6 (41.7–73.8)	32.9 (27.5–38.9)	48.4 (36.6–60.5)
Vegetable garden	36.4 (30.1–43.1)	9.9 (3.1–27.3)	20.3 (16.3–25.0)	16.9 (11.0–25.2)	28.0 (21.2–36.0)	36.7 (24.7–50.7)	28.6 (22.1–36.2)	39.3 (23.2–58.0)	36.2 (29.9–42.9)	23.4 (14.1–36.3)
Canteen and alternative food outlets	11.1 (7.4–16.4)	35.2 (20.5–53.4)	7.4 (5.1–10.6)	23.9 (17.7–31.3)	9.4 (5.8–15.0)	21.6 (12.9–33.8)	10.1 (6.0–16.6)	13.6 (5.3–30.7)	13.4 (9.5–18.5)	29.1 (19.0–41.7)
Canteen only	18.8 (14.2–24.4)	62.4 (44.5–77.4)	7.0 (4.9–9.7)	73.5 (64.8–80.7)	34.0 (26.6–42.4)	61.2 (46.3–74.2)	25.3 (18.8–33.0)	85.9 (69.1–94.3)	29.2 (23.7–35.3)	60.6 (46.9–72.7)
Alternative food outlets	27.4 (21.7–34.0)	0.1 (0.0–0.09)	41.1 (36.2–46.3)	0	10.9 (7.9–14.9)	0.4 (0.1–24.5)	11.2 (7.0–17.6)	0.0 (0.0–1.5)	11.6 (8.1–16.2)	1.9 (0.0–12.8)
Without canteen and alternative food outlets	42.7 (36.3–49.4)	1.1 (0.3–4.5)	44.5 (39.6–49.5)	2.6 (0.0–14.4)	45.7 (37.7–53.8)	13.1 (5.7–27.4)	53.4 (46.1–60.6)	0.0 (0.0–1.3)	45.9 (40.1–51.8)	8.4 (2.7–23.7)
Food and beverage availability										
School meals	99.1 (98.4–99.6)	23.7 (12.6–40.3)	96.8 (93.5–98.4)	20.3 (13.8–29.0)	98.8 (94.8–99.7)	29.8 (18.5–44.2)	95.7 (90.5–98.2)	25.9 (13.4–44.1)	97.9 (94.9–99.1)	27.3 (16.4–41.9)
Drinking water	95.4 (90.7–97.8)	100.0	95.3 (93.0–96.8)	99.7 (97.9–100.0)	99.8 (95.7–99.8)	100.0	99.1 (95.7–99.8)	100.0	99.9 (99.6–100.0)	100.0
Canteen food and beverage availability (considering only schools with canteens)										
Natural fruit juice/refreshments	74.4 (60.7–84.6)	99.2 (97.2–99.8)	54.5 (41.2–67.2)	91.0 (85.6–94.5)	77.1 (61.0–87.8)	88.5 (82.2–92.8)	73.7 (60.2–83.9)	89.3 (72.4–96.4)	61.3 (50.9–70.7)	91.0 (79.5–96.3)
Fresh fruit or fruit salad	14.2 (7.3–25.7)	34.9 (23.0–49.1)	16.4 (9.2–27.4)	48.2 (39.8–56.7)	6.3 (2.5–15.2)	47.3 (33.7–61.4)	3.3 (1.0–10.5)	38.7 (24.4–55.4)	12.7 (7.0–21.8)	65.0 (51.2–76.6)
Sweetened beverages (including soft drinks)	70.7 (58.9–80.2)	80.7 (65.3–90.3)	44.2 (31.3–58.0)	87.8 (81.6–92.1)	55.9 (41.6–69.2)	74.0 (60.1–84.3)	26.2 (18.4–35.7)	46.8 (30.0–64.3)	72.4 (63.6–79.7)	77.3 (66.2–85.6)
Packaged salty snacks	63.7 (51.9–74.1)	65.7 (53.0–76.5)	52.0 (37.7–66.1)	65.3 (56.6–73.1)	55.0 (40.1–69.1)	52.7 (39.2–65.8)	15.8 (8.2–28.3)	15.6 (7.4–29.9)	63.9 (53.6–73.1)	45.8 (34.3–57.8)
Sweet and savory crackers	43.5 (31.4–56.4)	51.4 (34.6–67.9)	40.4 (29.5–52.3)	61.2 (52.8–69.1)	35.2 (22.0–51.2)	59.2 (45.3–71.7)	20.6 (11.6–33.9)	53.6 (35.8–70.6)	45.9 (35.6–56.6)	42.5 (30.7–55.3)
Candies and others	50.8 (38.0–63.4)	46.4 (30.9–62.6)	49.8 (37.0–62.7)	62.8 (54.7–70.2)	28.2 (16.6–43.6)	63.0 (49.5–74.8)	14.1 (7.1–26.0)	19.4 (9.9–34.6)	50.4 (40.2–60.7)	46.6 (34.6–59.1)
Alternative food outlet food and beverage availability (considering only schools with alternative food outlets)										
Natural fruit juice/refreshment	69.3 (59.2–77.8)	53.0 (30.7–74.1)	43.2 (35.6–51.2)	41.7 (23.3–62.8)	40.0 (25.4–56.7)	38.1 (14.4–69.3)	29.5 (15.8–48.4)	63.6 (32.6–86.4)	35.5 (24.3–48.5)	38.2 (19.5–61.2)
Fresh fruits or fruit salad	10.0 (4.6–20.4)	14.3 (2.9–48.2)	11.4 (6.5–19.1)	17.0 (4.6–46.8)	-	1.3 (0.0–9.5)	5.5 (1.3–20.9)	37.6 (8.1–80.4)	3.6 (1.5–8.5)	22.0 (11.1–38.9)
Sweetened beverages (includes soft drinks)	68.9 (57.3–78.6)	60.0 (40.0–77.1)	73.9 (67.2–79.7)	80.8 (61.9–91.6)	76.7 (64.5–85.7)	65.6 (36.6–86.3)	84.1 (64.3–93.9)	41.7 (10.8–80.9)	67.9 (55.1–78.4)	63.2 (35.9–84.0)
Packaged salty snacks	68.4 (57.4–77.6)	55.5 (33.5–75.5)	72.3 (65.2–78.5)	64.1 (46.6–78.5)	71.3 (59.2–80.9)	55.8 (27.8–80.6)	73.7 (54.6–86.7)	58.9 (15.5–91.8)	67.9 (55.7–78.0)	50.2 (26.9–73.4)
Sweet and savory crackers	28.3 (19.2–39.6)	7.9 (1.2–36.7)	38.7 (31.7–46.2)	44.3 (25.7–64.7)	44.0 (28.9–60.2)	51.8 (24.0–78.5)	31.0 (16.5–50.7)	79.9 (31.3–97.2)	39.6 (27.0–53.8)	40.1 (19.5–64.8)
Candies and others	51.4 (40.0–62.6)	36.2 (19.6–56.8)	65.3 (58.0–71.9)	77.1 (63.2–86.9)	52.2 (35.9–68.1)	84.8 (59.7–95.4)	63.7 (44.8–79.2)	68.3 (25.7–93.1)	52.4 (40.4–64.1)	65.3 (38.7–84.9)

Private schools in all studied strata showed the greatest availability of physical activity structure/items. Sports courts and sports or games equipment were the most frequent items in public (69.2% and 90.7%, respectively) and private (94.1% and 99.8%) schools. Public schools in capitals showed a higher frequency of courts (80.2%) than schools outside capitals (66.5%). All Southeastern and Southern private schools analyzed had sports or games equipment. Note that running tracks and swimming pools are the rarest items, but the private sector showed a significantly higher frequency for swimming pools: 34.1% to 1.4% in the public sector (Tables 3 and 4).

**Table 3:** Characterization of school physical activity environments for all Brazilian schools according to location and administrative dependence. Brazil, 2015.

Variables	Brazil	Administrative dependence		Location			

				Capital		Other cities	
		
		Public	Private	Public	Private	Public	Private
						
	% 95%CI	% 95%CI	% 95%CI	% 95%CI	% 95%CI	% 95%CI	% 95%CI
Structures and materials availability							
Sports courts	72.8 (70.4–75.1)	69.2 (66.4–71.9)	94.1 (91.2–96.0)	80.2 (76.1–83.7)	95.8 (93.3–97.4)	66.5 (63.3–69.6)	92.8 (87.8–95.8)
Indoor sports courts (considering only schools that reported having a court)	83.9 (81.1–86.4)	83.3 (80.2–86.1)	86.5 (79.5–91.4)	81.0 (76.0–85.2)	94.0 (89.4–96.7)	84.0 (80.3–87.2)	80.6 (68.8–88.7)
Running/athletics tracks	2.5 (1.5–4.1)	1.6 (0.7–3.6)	7.8 (4.8–12.3)	1.4 (0.8–2.5)	6.9 (2.8–15.9)	1.7 (0.1–4.4)	8.4 (5.0–14.0)
Swimming pools	5.9 (4.7–7.5)	1.2 (0.7–1.9)	34.1 (27.3–41.7)	2.4 (0.9–6.0)	36.8 (29.3–44.9)	0.9 (0.5–1.5)	32.2 (21.8–44.6)
Halls	49.9 (46.4–53.3)	48.4 (44.5–52.3)	58.2 (50.6–65.4)	50.9 (44.6–57.2)	53.7 (45.2–62.0)	47.8 (43.3–52.4)	61.6 (50.0–72.1)
Sports or games equipment	92.0 (90.1–93.6)	90.7 (88.4–92.5)	99.8 (99.0–100.0)	92.9 (90.1–94.9)	99.5 (97.8–99.9)	90.1 (87.4–92.3)	100.0
Changing rooms	28.7 (25.7–32.0)	22.2 (19.0–25.7)	67.5 (60.6–73.8)	23.5 (19.2–28.5)	70.1 (62.3–76.8)	21.8 (18.1–26.1)	65.6 (54.4–75.3)

**Table 4:** Characterization of the school nutritional environment for all Brazilian schools according to location and administrative dependence. Brasil, 2015.

Variables	Region									

	North		Nordeste		North		Sul		North	
				
	Public	Privada	Public	Privada	Public	Privada	Public	Privada	Public	Privada
									
	% 95%CI	% 95%CI	% 95%CI	% 95%CI	% 95%CI	% 95%CI	% 95%CI	% 95%CI	% 95%CI	% 95%CI
Structures and materials availability										
Sports courts	51.4 (45.0–57.7)	85.7 (70.8–93.7)	38.0 (33.1–43.1)	90.4 (85.3–93.8)	88.2 (82.7–92.1)	96.6 (90.1–98.9)	84.9 (78.9–89.4)	96.8 (81.6–99.5)	74.8 (68.6–80.1)	95.3 (89.6–98.0)
Indoor sports courts (considering only schools that reported having a court)	87.0 (79.0–92.3)	92.3 (68.4–98.5)	66.2 (57.6–73.9)	86.3 (78.3–91.6)	90.5 (85.5–93.9)	84.0 (70.4–92.1)	75.6 (68.6–81.5)	92.9 (81.0–97.6)	78.1 (71.1–83.7)	92.1 (82.6–96.7)
Running/athletics tracks	1.4 (0.6–3.4)	14.0 (4.4–36.4)	0.3 (0.0–0.9)	5.2 (1.8–14.1)	1.9 (0.4–8.7)	4.9 (1.5–14.8)	3.3 (1.6–6.6)	23.4 (12.7–39.0)	2.6 (1.1–5.7)	13.9 (6.2–28.3)
Swimming pools	1.6 (0.7–3.8)	26.5 (15.6–41.2)	0.6 (0.1–2.3)	31.5 (24.8–39.1)	1.2 (0.5–2.9)	36.9 (24.5–51.3)	0.3 (0.0–1.9)	24.0 (11.6–43.1)	4.0 (1.9–8.1)	43.1 (30.6–56.6)
Halls	44.4 (37.9–51.2)	57.5 (42.6–71.2)	44.4 (39.4–49.5)	61.1 (52.2–69.4)	47.7 (39.8–55.7)	54.1 (40.4–67.1)	60.3 (52.7–67.5)	55.0 (37.7–71.2)	53.6 (46.8–60.3)	76.9 (64.2–86.1)
Sports or games equipment	85.2 (79.7–89.4)	97.2 (82.4–99.6)	88.0 (84.7–90.7)	99.9 (99.3–100.0)	92.3 (87.3–95.5)	100.0	93.6 (87.8–95.5)	100.0	93.6 (89.7–96.1)	99.7 (98.1–100.0)
Changing rooms	21.3 (16.3–27.4)	41.5 (26.7–57.9)	15.6 (12.1–19.9)	65.5 (57.1–73.0)	27.7 (21.3–35.1)	71.3 (57.7–81.9)	22.3 (16.3–29.7)	70.4 (52.3–83.7)	15.8 (11.5–21.4)	67.2 (55.5–77.1)

A very low internal consistency was observed for the Availability of production structure and offer of food and beverage score (alpha = 0.1804), and subsequent analyses ignored it.

The Food and beverage availability score showed an acceptable internal consistency (alpha = 0.79); item correlation ranged from -0.5522 to 0.7184. Though no school reached the maximum score, some public and private schools scored 85.7 points (12 desirable items). At least one private school scored only one desirable item (7.1 points), and public schools, two (14.3 points) (results not shown). Public schools (64.9; 95%CI 64;65.8) scored higher than private ones (55.8; 95%CI 53.4;58.2). The region, but not the municipality, influenced scores. Southern public schools scored significantly higher than Northern, Northeastern, and Midwestern ones, and its private schools, than Northern, Northeastern, and Southeastern ones, totaling the highest overall average. Both Northeastern administrative dependences scored the lowest among all regions (Table 5).

**Table 5:** Description of Food and beverage availability scores and of structures and materials for physical activity availability, according to administrative dependence and geographic variables. Brasil, 2015.

Variables	Food and beverages availability score		Structures and materials availability score	
	
	Mean	95%CI	Mean	95%CI
Brazil	63.6	62.7–64.4	44.7	43.5–45.9
Public schools	64.9	64.0–65.8	41.6	40.3–42.8
Location				
Capital	63.6	62.1–65.2	45.2	43.4–47.0
Other cities	65.3	64.2–66.3	40.7	39.2–42.2
Region				
North	64.3	62.8–65.8	35.7	33.1–38.4
Northeast	63.0	61.9–64.1	30.3	28.5–32.1
Southeast	65.9	63.9–67.9	48.4	46.0–50.8
South	67.7	66.2–69.2	47.0	44.6–49.4
Midwest	63.2	61.7–64.7	43.2	41.0–45.5
Private schools	55.8	53.4–58.2	63.3	60.6–66.0
Location				
Capital	53.7	51.5–55.9	64.8	62.4–67.2
Other cities	57.4	53.8–61.0	62.2	57.7–66.6
Region				
North	55.9	52.5–59.3	57.4	51.7–63.0
Northeast	52.7	50.6–54.7	61.6	58.2–64.9
Southeast	55.7	51.4–60.0	63.6	58.6–68.5
South	64.2	61.0–67.4	66.0	59.7–72.3
Midwest	59.0	55.7–62.3	69.1	64.3–74.0

^a^ The Food and beverage availability score consists of the following items: school meals offer; availability of drinking water; and, in the case of the presence of canteens and alternative food outlets, the offer of: 1) Natural fruit juice or refreshment; 2) fresh fruits or fruit salad; 3) sweetened beverages (including soft drink); 4) industrialized packaged salty snacks; 5) savory or sweet crackers; and 6) candy and others.

^b^ The Structures and materials for physical activity availability score consists of the following items: sports courts; at least one indoor sports court; running/athletics tracks; halls; swimming pools; changing rooms; sports or games equipment.

The Structures and materials availability score had an internal consistency below acceptable (alpha = 0.6), and item correlation ranged from -0.011 to 0.798. Both administrative dependences showed extreme maximum values, and we observed schools with all seven desirable structures (100 points). However, a few public schools lacked any structures, whereas private schools had at least one available (results not shown). Private schools showed a higher average score (63.3; 95%CI 60.6;66) than public schools (41.6; 95%CI 40.3;42.8). Public school scores related to regions and municipalities, whereas private schools varied only across regions. Public schools in the Southeast scored significantly higher than in the North, Northeast and Midwest, and in the South, significantly higher than in the North and Northeast, whereas the Midwest showed the best private school environment. Public schools in capitals scored higher than those outside capitals (Table 5).

## DISCUSSION

Using data from public and private schools in municipalities from the five Brazilian macro-regions, we found, via indicators, a healthier food environment in public schools, and a better physical activity environment in private schools. However, considering that healthy eating and regular physical activity reduce the risks of non-communicable diseases, and that the school environment should promote healthy practices to prevent these diseases^24^, Brazilian public and private school environments still need improvement.

We found significant differences in the food environment of public and private schools – the most evident being school meal offer, the presence of usable kitchens (allowing meal preparation) and canteens, and the types of food canteens sold.

PNAE guarantees universal meal offer to elementary students^5^in public schools, justifying our results. PNAE is an important element of the school food environment, since the frequent consumption of the food it offers relates to a better nutrition for Brazilian students^14^. Furthermore, the restriction of PNAE to public schools may contribute to the high prevalence of canteens in private schools^25^. Its low frequency in public schools probably contributed to their higher scores.

The results of this study converge with the literature on the subject. Data from the Study of Cardiovascular Risks in Adolescents (Erica), conducted with 1247 schools in 124 municipalities, found a higher availability of ultra-processed foods in private schools, which most often had alternative food outlets in and around them^8^. Another study conducted in Santos, in the state of São Paulo, evaluated the food environment in the school surroundings in the city and found that the outlets closest to the schools offered a greater amount of ultra-processed foods^26^.

Knowing the Brazilian school food environment is important, since the literature indicates that the presence of canteens and vending machines in and around schools offering a greater availability of ultra-processed foods strongly influence the chances of students consuming them^11^. Also note that, despite the absence of national regulation, state and municipal laws and regulations aim to control and/or prohibit the availability of unhealthy foods in schools^4,7,15,27^. However, the frequency of their commercialization is still high.

Private schools showed more favorable structures/items for physical activity, with a higher frequency of swimming pools, running tracks, and changing rooms than the public sector (32.9%, 6.2%, and 45.3% more, respectively), an expressive difference. Most Brazilian schools had halls and sports or games materials, spaces and items essential for the functioning of schools and physical education classes. Still, they may fall short of promoting physical activity, since more available structures/items result in a greater likelihood of more intense physical activity among students^10^.

Evidence points to the relation between the presence of these structures and physical activity levels. A Canadian study significantly associated physical activity with baseball fields, covered gyms, school size, and others^17^. Moreover, studies conducted in Sweden, Belgium, and the United States found that better physical structures can significantly increase students’ physical activity levels^28,29^.

As for the national literature, a study in the city of Londrina, in the state of Paraná, conducted with 1562 students from 20 preschools found that covered halls were a common structure, but all schools lacked extracurricular physical activities and physical education classes, and less than a third had recreation rooms, parks, and portable toys^30^. Data analysis from PeNSE 2012, which included 109.104 Brazilian students from 2842 schools, showed that most schools were public, had at least one multi-sport court, and offered extracurricular sports activities (64%), whereas teacher-guided physical activities in school halls, usable swimming pools, and changing rooms were less common. Furthermore, schools with at least one physical activity structure/facility resulted in a higher chance of students participating in physical education, but increasing their physical activity during leisure time and the total level of physical activity, required at least four and two additional structures/facilities, respectively. Moreover, extracurricular sports activities in schools were positively associated with the level of physical activity and leisure time practice^10^.

The scores developed in this study contribute to the literature on school food and physical activity environments, exploring some of its dimensions via variables collected in a large national periodical survey.

In evaluating the internal consistency of indicators, the literature considers a 0.7 or above Cronbach’s alpha acceptable. In our study, Food and beverage availability scored 0.79. However, the Structures and materials availability for physical activities scored 0.6, partly because the indicator consists of a limited number of items^22,23^. Moreover, indicators assessing environments are difficult to estimate, and any sets of variables will fail to cover it perfectly, rendering values up to 0.6 acceptable^31^. In any case, we should interpret these indicators with caution, since all items had the same weight, regardless of their positivity, negativity, and frequency. Despite the importance of Availability of production structure and offer of food and beverage for an expanded evaluation of the school food environment, its score showed a very low consistency, probably due to the few items available in PeNSE. Thus, we chose to keep the description of the isolated variables, but omit their score values.

We excluded 93 schools from food environment analyses, and six, from physical activity environment analyses due to missing data on the studied variables. We performed sensitivity analyses considering missing values as absences of desirable items and found no differences between the results. Considering the size of our sample and the sensitivity analyses, such exclusions failed to impact our results.

We should highlight some strengths of this study. The descriptive approach of the environments of interest in a representative sample of schools in the five Brazilian macro-regions, together with the scores constructed from the PeNSE questionnaire, may be considered an innovation in evaluating these environments. Considering that PeNSE takes place periodically, evaluating the scores and describing each item in them may contribute to monitoring the Brazilian school environment, though we need to improve score validation.

Our findings indicate that schools are a space populated by several elements that can contribute to diet and physical activity. We conclude that public schools provide a more favorable food environment, whereas private schools, a physical activity environment, though we observed important differences, mainly among macro-regions. These findings reinforce the importance of encouraging the adoption of health promotion practices and policies in schools.
