# Supplementary material for: Characteristics of Brazilian school food and physical activity environments: PeNSE 2015
Source: Rev Saude Publica. 2021 Dec 16;55:115. doi: 10.11606/s1518-8787.2021055003377 (PMC8687607; doi:10.11606/s1518-8787.2021055003377)
Supplement: Translation

# Translation: Características do ambiente escolar relativas à alimentação e atividade física: PeNSE 2015

**Authors:** Lucyane Barbosa Oliveira Souza, Ana Beatriz Coelho de Azevedo, Daniel Henrique Bandoni, Daniela Silva Canella

**Affiliations:** I Brasil Universidade do Estado do Rio de Janeiro. Programa de Pós-Graduação em Alimentação, Nutrição e Saúde. Rio de Janeiro, RJ, Brasil; II Brasil Universidade Federal de São Paulo. Instituto de Saúde e Sociedade. Departamento Saúde, Clínica e Instituições. Santos, SP, Brasil; III Brasil Universidade do Estado do Rio de Janeiro. Instituto de Nutrição. Departamento de Nutrição Aplicada. Rio de Janeiro, RJ, Brasil

**Keywords:** Alimentação Escolar, Exercício Físico, Ensino Fundamental e Médio, Serviços de Saúde Escolar, Promoção da Saúde

## Abstract

**OBJETIVO:**

Caracterizar o ambiente alimentar e o ambiente para a prática de atividade física em escolas brasileiras públicas e privadas, bem como desenvolver indicadores-síntese de avaliação desses ambientes.

**MÉTODOS:**

Estudo transversal, realizado com dados oriundos de questionário sobre características da escola da Pesquisa Nacional de Saúde do Escolar 2015 respondido pelo diretor ou responsável pelas escolas, referentes a 3.040 escolas públicas e privadas de todo o território nacional. As variáveis relacionadas aos ambientes alimentar e de prática de atividades físicas foram descritas isoladamente e, adicionalmente, foi desenvolvido um indicador-síntese para cada um dos ambientes, com pontuação variando de 0 a 100. A frequência de cada variável e a pontuação média dos escores foi descrita segundo a esfera administrativa (pública ou privada).

**RESULTADO:**

A oferta de alimentação escolar foi predominante na rede pública (97,8%) e a presença de cantinas na rede privada (89,8%). A presença de pontos alternativos de venda foi similar nas duas esferas. A disponibilidade de todos os marcadores de alimentação saudável e não saudável nas cantinas foi mais frequente nas escolas privadas. A pontuação do escore “Disponibilidade de alimentos e bebidas” foi maior para as escolas públicas (64,9), comparada à das privadas (55,8). A caraterização do ambiente para a prática de atividade física mostrou que a presença de quadra de esporte e de material esportivo foi frequente em escolas públicas (69,2% e 90,7%, respectivamente) e em escolas privadas (94,1% e 99,8%, respectivamente), ainda que em frequência significativamente maior no segundo grupo. A pontuação do escore “Disponibilidade de estruturas e materiais” foi maior entre escolas privadas do que entre as públicas (63,3 e 41,6, respectivamente).

**CONCLUSÕES:**

O ambiente alimentar de escolas públicas foi mais favorável à promoção da alimentação saudável que o de escolas privadas, sendo que o oposto foi verificado para o ambiente para a prática de atividade física.

## INTRODUÇÃO

As doenças crônicas não transmissíveis (DCNT) são um dos maiores problemas de saúde pública no Brasil e no mundo, estando a alimentação não saudável e a inatividade física entre seus principais fatores de risco^1^. Comportamentos adquiridos na infância e na adolescência tendem a se perpetuar na vida adulta com consequências para a qualidade de vida, o que torna a promoção da saúde essencial nesta fase^2^.

O ambiente escolar, por ser um espaço de socialização e interação, é reconhecido por organismos internacionais^3^ e em políticas públicas brasileiras^4^ como um local estratégico para ações de promoção da saúde. A implementação dessas ações pode contribuir para uma alimentação adequada e saudável e para a prática regular de atividade física. No Brasil, em relação ao ambiente alimentar, as escolas públicas têm oferta universal de alimentação garantida pelo Programa Nacional de Alimentação Escolar (PNAE)^5^, e podem ter cantinas (reguladas ou não pelos governos locais^7,8^) e venda de alimentos em seu entorno^8^. As escolas privadas não contam com o PNAE, mas podem ter oferta de refeições e, além de cantina e venda no entorno, máquinas de autosserviços^8^. Quanto à atividade física, a disciplina de educação física é parte do currículo escolar^9^, e juntamente com as estruturas/instalações existentes e a realização de atividades extracurriculares nas escolas^10,11^, constituem uma janela de oportunidade para a adesão às práticas de atividade física.

Evidências apontam a influência do ambiente escolar, avaliado principalmente por meio da disponibilidade de alimentos e de bebidas e da presença de estruturas e de atividades, na alimentação^12^ e na prática de atividade física^10,16,17^ de crianças e adolescentes. No contexto nacional, sinalizam ainda diferenças importantes entre escolas públicas e privadas e entre as regiões brasileiras^8,18^. A Pesquisa Nacional de Saúde do Escolar (PeNSE) contempla a avaliação de aspectos do ambiente escolar relacionados à alimentação e à prática de atividade física. Explorar tais aspectos e indicadores-síntese construídos a partir dessas variáveis, bem como suas diferenças a depender da localização das escolas, em um estudo com dados representativos do país, pode contribuir para o conhecimento do ambiente escolar, seu monitoramento e para a formulação de ações de promoção de saúde na escola.

Diante disso, o objetivo do presente estudo foi caracterizar o ambiente alimentar e o ambiente para a prática de atividade física em escolas públicas e privadas brasileiras, bem como desenvolver indicadores-síntese de avaliação desses ambientes.

## MÉTODOS

Trata-se de estudo transversal utilizando dados da PeNSE 2015, realizada pelo Instituto Brasileiro de Geografia e Estatística (IBGE) com os Ministérios da Saúde e da Educação.

Foram utilizados dados da Amostra 1 da PeNSE 2015, que contempla escolas com turmas de 9º ano do ensino fundamental e envolveu 3.040 escolas públicas e privadas, distribuídas em 675 municípios brasileiros. A amostra foi dimensionada para estimar os parâmetros populacionais (prevalência ou proporção) em diferentes domínios geográficos brasileiros: 26 capitais e o Distrito Federal, 26 Unidades da Federação (abrangendo os demais municípios, à exceção das capitais), cinco macrorregiões e o Brasil, totalizando 53 estratos geográficos.

A seleção das escolas em cada estrato foi feita a partir do Censo Escolar 2013, com probabilidade de seleção proporcional à quantidade de turmas do 9º ano. A coleta de dados foi realizada utilizando questionário eletrônico, seguindo a metodologia da Global School-Based Student Health Survey, desenvolvida pela Organização Mundial da Saúde (OMS), sendo os módulos de avaliação do ambiente (objeto de interesse deste estudo) aplicados somente aos diretores ou responsáveis pela escola^19^.

Para caracterização do ambiente alimentar escolar foram consideradas variáveis referentes à: presença de cozinha em condições de uso; presença de refeitório em condições de uso; presença de horta; oferta de alimentação escolar (merenda escolar/almoço); disponibilidade de água potável; presença de cantina; presença de ponto de venda alternativo na entrada ou entorno da escola; no caso de presença de cantina e de ponto alternativo, avaliou-se a oferta de: 1) suco ou refresco natural de fruta; 2) frutas frescas ou salada de frutas; 3) bebidas adoçadas (incluindo refrigerante); 4) salgadinhos industrializados vendidos em pacotes; 5) biscoito ou bolachas salgados ou doces; e 6) balas, confeitos, doces e outros.

Na avaliação do ambiente para a prática de atividade física, foi considerada a presença de: quadra de esporte; pelo menos uma quadra de esporte coberta; pista de corrida/atletismo; pátio; piscina; vestiários; material esportivo ou para jogos e brincadeiras.

As variáveis disponíveis no questionário da PeNSE permitem estabelecer um diálogo entre este estudo e dimensões de modelos teórico-conceituais sobre ambiente alimentar e ambiente para a prática de atividade física. Considerando a escola como um ambiente alimentar organizacional, as variáveis disponíveis da PeNSE expressam elementos do âmbito institucional (presença de cozinha, refeitório, horta, cantina e ponto alternativo de venda) e, no âmbito dos estabelecimentos, da dimensão de disponibilidade^20^. Em relação à atividade física, as variáveis representam elementos da dimensão de disponibilidade do ambiente externo construído^21^.

Considerando essas dimensões, foram criados três indicadores-síntese: dois escores para o ambiente alimentar (“Disponibilidade de estruturas de produção e oferta de alimentos e bebidas” e “Disponibilidade de alimentos e bebidas”) e um escore para ambiente para a prática de atividade física (“Disponibilidade de estruturas e materiais”). Para tanto, todas as variáveis foram dicotomizadas em 1 e 0, assumindo pontuação 1 para os itens desejáveis (ex.: oferta de alimentação escolar, ausência de oferta de refrigerante na cantina e presença de pátio) e 0 para os itens não desejáveis (ex.: oferta de bebida açucarada na cantina e ausência de material esportivo). No caso de uma escola sem cantina, por exemplo, foi atribuída pontuação 1 para a disponibilidade dos itens não saudáveis, por não oferecê-los, e 0 para a disponibilidade dos itens saudáveis pela mesma razão.

O escore *Disponibilidade de estruturas de produção e oferta de alimentos e bebidas* contemplava cinco itens: presença de cozinha, refeitório, horta, cantina e ponto alternativo de venda; o escore *Disponibilidade de alimentos e bebidas*, 14 itens: oferta de alimentação escolar; disponibilidade de água potável; no caso de presença de cantina e de ponto alternativo, avaliou-se em cada um destes a oferta de: 1) suco ou refresco natural de fruta, 2) frutas frescas ou salada de frutas, 3) bebidas adoçadas (incluindo refrigerante), 4) salgadinhos industrializados vendidos em pacotes, 5) biscoito ou bolachas salgadas ou doces, e 6) balas, confeitos, doces e outros; e o escore *Disponibilidade de estruturas e materiais*, sete itens: quadra de esporte; pelo menos uma quadra de esporte coberta; pista de corrida/atletismo; pátio; piscina; vestiários; material esportivo ou para jogos e brincadeiras. A pontuação dos escores podia variar de 0 a 5, 0 a 14 e 0 a 7, respectivamente. Posteriormente, os escores foram padronizados na escala de 0–100 pontos. A consistência interna de cada escore foi avaliada pelo coeficiente de alfa de Cronbach, sendo considerada aceitável alfa > 0,7^22,23^, e pela correlação entre os itens utilizando o coeficiente de correlação de Pearson.

Adicionalmente, foram utilizadas nas análises variáveis referentes a aspectos geográficos, como macrorregiões e localização das escolas (capitais ou não capitais), e dependência administrativa (pública ou privada).

Foram feiras as análises descritivas com apresentação de frequência e de intervalo de confiança de 95% (IC95%) de cada uma das variáveis. As análises foram realizadas para o Brasil como um todo, as cinco macrorregiões e a localização das escolas, sendo todas estratificadas considerando a dependência administrativa das escolas. Para os escores, foram calculados valores médios de pontuação e IC95% segundo variáveis geográficas e dependência administrativa da escola. A ausência de sobreposição entre os intervalos de confiança foi assumida como uma diferença significativa.

Foi utilizado o software estatístico Stata SE versão 15.1 (Stata Corp., College Station, USA), empregando o modulo *survey*, que considera os efeitos do plano amostral complexo da pesquisa.

A PeNSE foi aprovada na Comissão Nacional de Ética em Pesquisa (Conep), do Conselho Nacional de Saúde, por meio do parecer Conep n. 1006467, de 30 de março de 2015.

## RESULTADOS

Foram analisados dados de 2.947 escolas brasileiras em relação ao ambiente alimentar e de 3.034 em relação ao ambiente para a prática de atividade física, sendo cerca de 80% dessas escolas públicas e 20% privadas. Os resultados de caracterização do ambiente alimentar escolar segundo a dependência administrativa mostraram que a oferta de alimentação escolar e a presença de cozinha em condições de uso foi predominante nas escolas públicas (97,8% e 96,7% contra 26,2% e 74,9% nas escolas privadas, respectivamente), enquanto a presença de cantina foi mais frequente nas escolas privadas (89,8% contra 33,0%). A disponibilidade de água potável foi verificada em mais de 98% das escolas. Os pontos alternativos de venda foram mais frequentes nas escolas públicas (30,4% contra 25,2%) (Tabela 1).

**Tabela 1:** Caracterização do ambiente alimentar escolar para o conjunto das escolas brasileiras e segundo localização e dependência administrativa. Brasil, 2015.

Variáveis	Brasil	Dependência Administrativa		Localização			

				Capital		Não capital	
		
		Público	Privada	Público	Privada	Público	Privada
						
	% (IC95%)	% (IC95%)	% (IC95%)	% (IC95%)	% (IC95%)	% (IC95%)	% (IC95%)
Presença de estruturas de produção e oferta de alimentos e bebidas							
Cozinha em condições de uso	93,6 (92,3–94,7)	96,7 (95,6–97,6)	74,9 (69,0–80,0)	98,2 (97,3–98,8)	73,1 (64,8–80,1)	96,4 (95,0–97,4)	76,2 (67,7–83,0)
Refeitório em condições de uso	60,8 (57,8–63,6)	61,0 (57,6–64,2)	59,6 (52,9–66,0)	71,0 (65,6–75,8)	62,9 (54,8–70,4)	58,5 (54,6–62,3)	57,1 (48,2–65,6)
Horta	27,6 (24,5–30,9)	27,4 (24,0–31,2)	28,6 (22,0–36,2)	22,7 (18,0–28,3)	22,0 (15,1–30,9)	28,6 (24,5–33,0)	33,6 (23,7–45,2)
Cantina e ponto alternativo de venda	11,4 (9,43–13,6)	9,4 (7,4–11,9)	22,9 (20,1–27,5)	12,2 (8,6–17,0)	33,4 (25,3–42,7)	8,7 (6,5–11,7)	15,0 (8,9–24,1)
Apenas cantina	29,8 (26,6–33,3)	23,6 (20,1–27,5)	66,8 (58,9–73,9)	22,9 (18,0–28,8)	65,2 (56,0–73,3)	23,7 (19,7–28,3)	68,1 (55,3–78,7)
Apenas ponto alternativo de venda	18,3 (16,3–20,5)	21,0 (18,7–23,5)	2,3 (0,4–11,9)	19,7 (15,3–25,1)	0,0 (0,0–0,2)	21,3 (18,8–24,0)	3,7 (0,1–21,1)
Sem cantina e sem ponto alternativo de venda	40,5 (37,1–44,0)	46,0 (42,2–49,9)	8,0 (3,9–15,5)	45,2 (39,2–51,4)	0,1 (0,0–0,3)	46,2 (41,7–50,9)	13,3 (6,3–25,6)
Disponibilidade de alimentos e bebidas na escola							
Alimentação escolar	87,5 (85,5–89,3)	97,8 (96,4–98,7)	26,2 (19,7–34,0)	98,2 (97,0–99,0)	25,1 (18,2–33,6)	97,7 (95,9–98,8)	27,0 (17,5–39,1)
Água potável	98,3 (97,6–98,8)	98,0 (97,2–98,6)	99,9 (99,4–100,0)	99,4 (99,0–99,7)	100,0	97,7 (96,7–98,4)	99,8 (98,9–100,0)
Disponibilidade de alimentos e bebidas na cantina *(considerando apenas escolas com cantina)*							
Suco/refresco natural de frutas	78,5 (72,9–83,2)	72,2 (63,8–79,2)	90,2 (86,6–92,9)	77,8 (69,3–84,4)	91,2 (86,6–94,3)	70,5 (60,0–79,2)	89,4 (83,9–93,1)
Frutas frescas ou salada de fruta	22,1 (18,4–26,4)	8,5 (5,5–12,7)	47,4 (40,3–54,6)	16,9 (9,2–29,2)	56,5 (48,1–64,5)	6,0 (3,4–10,4)	39,3 (28,4–51,3)
Bebidas adoçadas (inclui refrigerantes)	61,4 (55,7–66,9)	53,3 (45,1–61,2)	76,5 (69,5–82,3)	73,1 (62,4–81,7)	81,1 (76,3–85,0)	47,6 (38,1–57,2)	72,4 (60,3–81,9)
Salgadinho de pacote	51,5 (45,6–57,4)	50,5 (42,1–58,8)	53,5 (46,4–60,5)	61,9 (51,3–71,5)	58,3 (49,9–66,3)	47,2 (37,2–57,4)	49,2 (38,6–55,9)
Biscoitos e bolachas	43,3 (37,3–49,5)	35,5 (27,7–44,2)	57,6 (50,4–64,4)	40,6 (29,5–52,8)	60,1 (51,4–68,2)	34,1 (24,7–44,8)	55,3 (44,3–65,9)
Balas, confeitos, doces e outros	41,4 (35,8–47,2)	33,1 (25,8–41,3)	56,6 (49,7–63,3)	43,5 (31,7–56,1)	58,8 (50,7–66,5)	30,1 (21,7–40,1)	54,6 (44,2–64,7)
Disponibilidade de alimentos e bebidas em ponto alternativo de venda *(considerando apenas escolas com ponto alternativo de venda)*							
Suco/refresco natural de frutas	43,8 (38,2–49,5)	44,1 (38,3–50,1)	41,5 (26,8–57,9)	43,1 (32,6–54,2)	33,8 (18,6–53,3)	44,3 (37,5–51,4)	51,9 (23,4–79,1)
Frutas frescas ou salada de fruta	7,4 (5,1–10,8)	7,1 (4,6–10,8)	10,2 (4,9–20,2)	4,6 (2,8–7,4)	13,8 (6,1–28,3)	7,7 (4,7–12,4)	5,5 (1,3–20,1)
Bebidas adoçadas (inclui refrigerantes)	73,8 (69,2–77,8)	74,6 (69,7–78,9)	68,0 (52,6–80,2)	83,1 (74,6–89,2)	58,6 (39,1–75,7)	72,4 (66,8–77,4)	80,7 (57,3–92,9)
Salgadinho de pacote	69,7 (65,1–74,0)	71,4 (66,4–75,9)	57,7 (42,2–71,8)	78,1 (69,3–84,8)	56,5 (37,3–73,9)	69,7 (63,9–74,9)	59,2 (33,2–81,0)
Biscoitos e bolachas	39,2 (33,9–44,8)	38,2 (32,5–44,2)	46,4 (30,6–62,9)	40,0 (29,2–51,9)	42,6 (24,7–62,7)	37,7 (31,2–44,7)	51,5 (24,8–77,4)
2Balas, confeitos, doces e outros	61,0 (55,5–66,3)	58,9 (52,9–64,7)	76,2 (64,1–85,1)	54,8 (43,2–65,8)	80,7 (70,3–88,0)	60,0 (52,8–66,7)	70,1 (43,1–87,9)

A disponibilidade de frutas frescas e saladas de frutas foi significativamente mais frequente nas cantinas de escolas privadas (47,4%) quando comparadas às públicas (8,5%). A disponibilidade de tais itens foi baixa em pontos alternativos de venda, sem diferença entre dependência administrativa. Suco/refresco natural de fruta e bebidas adoçadas foram os itens mais frequentemente disponíveis nas cantinas das escolas privadas e das públicas (90,2% e 72,2%, respectivamente). Nos pontos alternativos de venda de escolas públicas, os itens mais frequentemente comercializados foram bebidas adoçadas (74,6%) e salgadinhos de pacote (71,4%), enquanto nas escolas privadas foram balas, confeitos, doces e outros (76,2%), bebidas adoçadas (68,0%) e salgadinhos de pacote (57,7%). A oferta de bebidas adoçadas nas cantinas das escolas públicas das cidades capitais foi significativamente superior em relação às cidades não capitais (73,1% contra 47,6%) (Tabela 1).

Sobre as regiões brasileiras, observou-se homogeneidade na elevada oferta de alimentação escolar e na presença de cozinha em condições de uso na rede pública. As regiões Sudeste e Nordeste foram as que tiveram, respectivamente, a maior (82,2% das escolas públicas e 71,9% das escolas privadas) e a menor frequência (32,4% das escolas públicas e 43,6% das escolas privadas) de refeitórios em condição de uso nas duas dependências administrativas. A presença de cantinas na rede pública foi menos frequente no Nordeste (14,4%), no entanto, esta região teve a maior disponibilidade de frutas frescas e salada de frutas nas cantinas existentes (16,4%). Um achado importante foi a ausência de oferta de frutas frescas e salada de frutas nos pontos alternativos de escolas públicas do Sudeste e a inexpressiva frequência de 1,3% na rede privada. Em relação à presença de horta, Norte e Centro-Oeste apresentaram maior diferença entre as escolas públicas (36,4% e 36,2%) e privadas (9,9% e 23,4%), e o Nordeste apresentou menor frequência entre as escolas públicas (20,3%) (Tabela 2).

**Tabela 2:** Caracterização do ambiente alimentar escolar segundo região e dependência administrativa. Brasil, 2015.

Variáveis	Região									

	Norte		Nordeste		Sudeste		Sul		Centro-Oeste	
				
	Público	Privada	Público	Privada	Público	Privada	Público	Privada	Público	Privada
									
	% IC95%	% IC95%	% IC95%	% IC95%	% IC95%	% IC95%	% IC95%	% IC95%	% IC95%	% IC95%
Presença de estruturas de produção e oferta de alimentos e bebidas										
Cozinha em condições de uso	95,1 (91,0–97,4)	74,2 (57,8–85,8)	94,5 (92,0–96,3)	63,5 (54,5–71,7)	99,3 (96,9–99,8)	80,7 (69,8–88,3)	94,7 (89,5–97,5)	86,3 (71,3–94,1)	95,8 (92,7–97,7)	69,2 (56,5–79,5)
Refeitório em condições de uso	61,0 (54,5–67,2)	52,3 (36,2–67,8)	32,4 (28,2–36,9)	43,6 (35,7–51,9)	82,2 (75,0–87,7)	71,9 (60,8–80,9)	68,1 (60,4–74,9)	58,6 (41,7–73,8)	32,9 (27,5–38,9)	48,4 (36,6–60,5)
Horta	36,4 (30,1–43,1)	9,9 (3,1–27,3)	20,3 (16,3–25,0)	16,9 (11,0–25,2)	28,0 (21,2–36,0)	36,7 (24,7–50,7)	28,6 (22,1–36,2)	39,3 (23,2–58,0)	36,2 (29,9–42,9)	23,4 (14,1–36,3)
Cantina e ponto alternativo de venda	11,1 (7,4–16,4)	35,2 (20,5–53,4)	7,4 (5,1–10,6)	23,9 (17,7–31,3)	9,4 (5,8–15,0)	21,6 (12,9–33,8)	10,1 (6,0–16,6)	13,6 (5,3–30,7)	13,4 (9,5–18,5)	29,1 (19,0–41,7)
Apenas cantina	18,8 (14,2–24,4)	62,4 (44,5–77,4)	7,0 (4,9–9,7)	73,5 (64,8–80,7)	34,0 (26,6–42,4)	61,2 (46,3–74,2)	25,3 (18,8–33,0)	85,9 (69,1–94,3)	29,2 (23,7–35,3)	60,6 (46,9–72,7)
Apenas ponto alternativo de venda	27,4 (21,7–34,0)	0,1 (0,0–0,09)	41,1 (36,2–46,3)	0	10,9 (7,9–14,9)	0,4 (0,1–24,5)	11,2 (7,0–17,6)	0,0 (0,0–1,5)	11,6 (8,1–16,2)	1,9 (0,0–12,8)
Sem cantina e sem ponto alternativo de venda	42,7 (36,3–49,4)	1,1 (0,3–4,5)	44,5 (39,6–49,5)	2,6 (0,0–14,4)	45,7 (37,7–53,8)	13,1 (5,7–27,4)	53,4 (46,1–60,6)	0,0 (0,0–1,3)	45,9 (40,1–51,8)	8,4 (2,7–23,7)
Disponibilidade de alimentos e bebidas na escola										
Alimentação escolar	99,1 (98,4–99,6)	23,7 (12,6–40,3)	96,8 (93,5–98,4)	20,3 (13,8–29,0)	98,8 (94,8–99,7)	29,8 (18,5–44,2)	95,7 (90,5–98,2)	25,9 (13,4–44,1)	97,9 (94,9–99,1)	27,3 (16,4–41,9)
Água potável	95,4 (90,7–97,8)	100,0	95,3 (93,0–96,8)	99,7 (97,9–100,0)	99,8 (95,7–99,8)	100,0	99,1 (95,7–99,8)	100,0	99,9 (99,6–100,0)	100,0
Disponibilidade de alimentos e bebidas na cantina *(considerando apenas escolas com cantina)*										
Suco/refresco natural de frutas	74,4 (60,7–84,6)	99,2 (97,2–99,8)	54,5 (41,2–67,2)	91,0 (85,6–94,5)	77,1 (61,0–87,8)	88,5 (82,2–92,8)	73,7 (60,2–83,9)	89,3 (72,4–96,4)	61,3 (50,9–70,7)	91,0 (79,5–96,3)
Frutas frescas ou salada de fruta	14,2 (7,3–25,7)	34,9 (23,0–49,1)	16,4 (9,2–27,4)	48,2 (39,8–56,7)	6,3 (2,5–15,2)	47,3 (33,7–61,4)	3,3 (1,0–10,5)	38,7 (24,4–55,4)	12,7 (7,0–21,8)	65,0 (51,2–76,6)
Bebidas adoçadas (inclui refrigerantes)	70,7 (58,9–80,2)	80,7 (65,3–90,3)	44,2 (31,3–58,0)	87,8 (81,6–92,1)	55,9 (41,6–69,2)	74,0 (60,1–84,3)	26,2 (18,4–35,7)	46,8 (30,0–64,3)	72,4 (63,6–79,7)	77,3 (66,2–85,6)
Salgadinho de pacote	63,7 (51,9–74,1)	65,7 (53,0–76,5)	52,0 (37,7–66,1)	65,3 (56,6–73,1)	55,0 (40,1–69,1)	52,7 (39,2–65,8)	15,8 (8,2–28,3)	15,6 (7,4–29,9)	63,9 (53,6–73,1)	45,8 (34,3–57,8)
Biscoitos e bolachas	43,5 (31,4–56,4)	51,4 (34,6–67,9)	40,4 (29,5–52,3)	61,2 (52,8–69,1)	35,2 (22,0–51,2)	59,2 (45,3–71,7)	20,6 (11,6–33,9)	53,6 (35,8–70,6)	45,9 (35,6–56,6)	42,5 (30,7–55,3)
Balas, confeitos, doces e outros	50,8 (38,0–63,4)	46,4 (30,9–62,6)	49,8 (37,0–62,7)	62,8 (54,7–70,2)	28,2 (16,6–43,6)	63,0 (49,5–74,8)	14,1 (7,1–26,0)	19,4 (9,9–34,6)	50,4 (40,2–60,7)	46,6 (34,6–59,1)
Disponibilidade de alimentos e bebidas em ponto alternativo de venda *(considerando apenas escolas com ponto alternativo de venda)*										
Suco/refresco natural de frutas	69,3 (59,2–77,8)	53,0 (30,7–74,1)	43,2 (35,6–51,2)	41,7 (23,3–62,8)	40,0 (25,4–56,7)	38,1 (14,4–69,3)	29,5 (15,8–48,4)	63,6 (32,6–86,4)	35,5 (24,3–48,5)	38,2 (19,5–61,2)
Frutas frescas ou salada de fruta	10,0 (4,6–20,4)	14,3 (2,9–48,2)	11,4 (6,5–19,1)	17,0 (4,6–46,8)	-	1,3 (0,0–9,5)	5,5 (1,3–20,9)	37,6 (8,1–80,4)	3,6 (1,5–8,5)	22,0 (11,1–38,9)
Bebidas adoçadas (inclui refrigerantes)	68,9 (57,3–78,6)	60,0 (40,0–77,1)	73,9 (67,2–79,7)	80,8 (61,9–91,6)	76,7 (64,5–85,7)	65,6 (36,6–86,3)	84,1 (64,3–93,9)	41,7 (10,8–80,9)	67,9 (55,1–78,4)	63,2 (35,9–84,0)
Salgadinho de pacote	68,4 (57,4–77,6)	55,5 (33,5–75,5)	72,3 (65,2–78,5)	64,1 (46,6–78,5)	71,3 (59,2–80,9)	55,8 (27,8–80,6)	73,7 (54,6–86,7)	58,9 (15,5–91,8)	67,9 (55,7–78,0)	50,2 (26,9–73,4)
Biscoitos e bolachas	28,3 (19,2–39,6)	7,9 (1,2–36,7)	38,7 (31,7–46,2)	44,3 (25,7–64,7)	44,0 (28,9–60,2)	51,8 (24,0–78,5)	31,0 (16,5–50,7)	79,9 (31,3–97,2)	39,6 (27,0–53,8)	40,1 (19,5–64,8)
Balas, confeitos, doces e outros	51,4 (40,0–62,6)	36,2 (19,6–56,8)	65,3 (58,0–71,9)	77,1 (63,2–86,9)	52,2 (35,9–68,1)	84,8 (59,7–95,4)	63,7 (44,8–79,2)	68,3 (25,7–93,1)	52,4 (40,4–64,1)	65,3 (38,7–84,9)

Referente ao ambiente para prática de atividade física escolar, a maior disponibilidade de estrutura/itens foi observada nas escolas privadas em todos os estratos estudados. Quadra de esportes e material esportivo foram os itens mais frequentes em escolas públicas (69,2% e 90,7%, respectivamente) e privadas (94,1% e 99,8%, respectivamente), sendo que as escolas públicas localizadas em capitais apresentaram maior frequência de quadras (80,2%) quando comparadas as de não capitais (66,5%). Nas escolas da rede privada das regiões Sudeste e Sul, o item material esportivo foi observado em todas as escolas analisadas. Os itens observados com menor frequência foram pista de corrida e piscina, contudo, a rede privada apresentou frequência significativamente maior para piscina, com 34,1%, comparado a 1,4% da rede pública (Tabelas 3 e 4).

**Tabela 3:** Caracterização do ambiente escolar para a prática de atividade física para o conjunto das escolas brasileiras e segundo localização e dependência administrativa. Brasil, 2015.

Variáveis	Brasil	Dependência Administrativa		Localização			

				Capital		Não capital	
		
		Público	Privada	Público	Privada	Público	Privada
						
	% (IC95%)	% (IC95%)	% (IC95%)	% (IC95%)	% (IC95%)	% (IC95%)	% (IC95%)
Disponibilidade de estruturas e materiais							
Quadra de esporte	72,8 (70,4–75,1)	69,2 (66,4–71,9)	94,1 (91,2–96,0)	80,2 (76,1–83,7)	95,8 (93,3–97,4)	66,5 (63,3–69,6)	92,8 (87,8–95,8)
Quadra de esporte coberta (considerando apenas escolas que relataram ter quadra)	83,9 (81,1–86,4)	83,3 (80,2–86,1)	86,5 (79,5–91,4)	81,0 (76,0–85,2)	94,0 (89,4–96,7)	84,0 (80,3–87,2)	80,6 (68,8–88,7)
Pista de corrida/ atletismo	2,5 (1,5–4,1)	1,6 (0,7–3,6)	7,8 (4,8–12,3)	1,4 (0,8–2,5)	6,9 (2,8–15,9)	1,7 (0,1–4,4)	8,4 (5,0–14,0)
Piscina	5,9 (4,7–7,5)	1,2 (0,7–1,9)	34,1 (27,3–41,7)	2,4 (0,9–6,0)	36,8 (29,3–44,9)	0,9 (0,5–1,5)	32,2 (21,8–44,6)
Pátio	49,9 (46,4–53,3)	48,4 (44,5–52,3)	58,2 (50,6–65,4)	50,9 (44,6–57,2)	53,7 (45,2–62,0)	47,8 (43,3–52,4)	61,6 (50,0–72,1)
Material esportivo	92,0 (90,1–93,6)	90,7 (88,4–92,5)	99,8 (99,0–100,0)	92,9 (90,1–94,9)	99,5 (97,8–99,9)	90,1 (87,4–92,3)	100,0
Vestiário	28,7 (25,7–32,0)	22,2 (19,0–25,7)	67,5 (60,6–73,8)	23,5 (19,2–28,5)	70,1 (62,3–76,8)	21,8 (18,1–26,1)	65,6 (54,4–75,3)

**Tabela 4:** Caracterização do ambiente escolar para a prática de atividade física segundo região e dependência administrativa. Brasil, 2015.

Variáveis	Região									

	Norte		Nordeste		Sudeste		Sul		Centro-Oeste	
				
	Público	Privada	Público	Privada	Público	Privada	Público	Privada	Público	Privada
									
	% IC95%	% IC95%	% IC95%	% IC95%	% IC95%	% IC95%	% IC95%	% IC95%	% IC95%	% IC95%
Disponibilidade de estruturas e materiais										
Quadra de esporte	51,4 (45,0–57,7)	85,7 (70,8–93,7)	38,0 (33,1–43,1)	90,4 (85,3–93,8)	88,2 (82,7–92,1)	96,6 (90,1–98,9)	84,9 (78,9–89,4)	96,8 (81,6–99,5)	74,8 (68,6–80,1)	95,3 (89,6–98,0)
Quadra de esporte coberta (considerando apenas escolas que relataram ter quadra)	87,0 (79,0–92,3)	92,3 (68,4–98,5)	66,2 (57,6–73,9)	86,3 (78,3–91,6)	90,5 (85,5–93,9)	84,0 (70,4–92,1)	75,6 (68,6–81,5)	92,9 (81,0–97,6)	78,1 (71,1–83,7)	92,1 (82,6–96,7)
Pista de corrida/atletismo	1,4 (0,6–3,4)	14,0 (4,4–36,4)	0,3 (0,0–0,9)	5,2 (1,8–14,1)	1,9 (0,4–8,7)	4,9 (1,5–14,8)	3,3 (1,6–6,6)	23,4 (12,7–39,0)	2,6 (1,1–5,7)	13,9 (6,2–28,3)
Piscina	1,6 (0,7–3,8)	26,5 (15,6–41,2)	0,6 (0,1–2,3)	31,5 (24,8–39,1)	1,2 (0,5–2,9)	36,9 (24,5–51,3)	0,3 (0,0–1,9)	24,0 (11,6–43,1)	4,0 (1,9–8,1)	43,1 (30,6–56,6)
Pátio	44,4 (37,9–51,2)	57,5 (42,6–71,2)	44,4 (39,4–49,5)	61,1 (52,2–69,4)	47,7 (39,8–55,7)	54,1 (40,4–67,1)	60,3 (52,7–67,5)	55,0 (37,7–71,2)	53,6 (46,8–60,3)	76,9 (64,2–86,1)
Material esportivo	85,2 (79,7–89,4)	97,2 (82,4–99,6)	88,0 (84,7–90,7)	99,9 (99,3–100,0)	92,3 (87,3–95,5)	100,0	93,6 (87,8–95,5)	100,0	93,6 (89,7–96,1)	99,7 (98,1–100,0)
Vestiário	21,3 (16,3–27,4)	41,5 (26,7–57,9)	15,6 (12,1–19,9)	65,5 (57,1–73,0)	27,7 (21,3–35,1)	71,3 (57,7–81,9)	22,3 (16,3–29,7)	70,4 (52,3–83,7)	15,8 (11,5–21,4)	67,2 (55,5–77,1)

Quantos aos indicadores-síntese, o escore Disponibilidade de estruturas de produção e oferta de alimentos e bebidas teve consistência interna muito baixa (alfa = 0,1804), não sendo, portanto, explorado nas análises subsequentes.

O escore Disponibilidade de alimentos e bebidas apresentou consistência interna aceitável (alfa = 0,79) e a correlação entre os itens variou de -0,5522 a 0,7184. Nenhuma das escolas atingiu a pontuação máxima, sendo que escolas públicas e privadas tiveram como valor máximo 85,7 pontos (12 itens desejáveis) e, quanto aos valores mínimos, ao menos uma escola privada apresentou somente um item desejável (7,1 pontos); nas escolas públicas, o valor mínimo foi de 14,3 pontos, equivalente a dois itens desejáveis (resultados não mostrados). As escolas públicas (64,9; IC95% 64,0;65,8) obtiveram maior pontuação do que as privadas (55,8; IC95% 53,4;58,2). A região, mas não a localização do município, foi relacionada ao escore. A região Sul se destacou com maior pontuação no escore de Disponibilidade de alimentos e bebidas, sendo este significativamente maior para as escolas públicas do que nas do Norte, Nordeste e Centro-Oeste, e para as privadas significativamente maior que no Norte, Nordeste e Sudeste. Nordeste apresentou a menor pontuação em ambas as dependências administrativas (Tabela 5).

**Tabela 5:** Descrição dos escores de disponibilidade de alimentos e bebidas e de disponibilidade de estruturas e materiais para atividade física, segundo dependência administrativa e variáveis geográficas. Brasil, 2015.

Variáveis	Escore Disponibilidade de alimentos e bebidas^a^		Escore Disponibilidade de estruturas e materiais^b^	
	
	Média	IC95%	Média	IC95%
Brasil	63,6	62,7–64,4	44,7	43,5–45,9
Escolas públicas	64,9	64,0–65,8	41,6	40,3–42,8
Localização				
Capital	63,6	62,1–65,2	45,2	43,4–47,0
Não capital	65,3	64,2–66,3	40,7	39,2–42,2
Região				
Norte	64,3	62,8–65,8	35,7	33,1–38,4
Nordeste	63,0	61,9–64,1	30,3	28,5–32,1
Sudeste	65,9	63,9–67,9	48,4	46,0–50,8
Sul	67,7	66,2–69,2	47,0	44,6–49,4
Centro-Oeste	63,2	61,7–64,7	43,2	41,0–45,5
Escolas privadas	55,8	53,4–58,2	63,3	60,6–66,0
Localização				
Capital	53,7	51,5–55,9	64,8	62,4–67,2
Não capital	57,4	53,8–61,0	62,2	57,7–66,6
Região				
Norte	55,9	52,5–59,3	57,4	51,7–63,0
Nordeste	52,7	50,6–54,7	61,6	58,2–64,9
Sudeste	55,7	51,4–60,0	63,6	58,6–68,5
Sul	64,2	61,0–67,4	66,0	59,7–72,3
Centro-Oeste	59,0	55,7–62,3	69,1	64,3–74,0

^a^ O escore de disponibilidade de alimentos e bebidas é composto pelos seguintes itens: oferta de alimentação escolar; disponibilidade de água potável; no caso de presença de cantina e de ponto alternativo, avaliou-se a oferta de: 1) suco ou refresco natural de fruta; 2) frutas frescas ou salada de frutas; 3) bebidas adoçadas (incluindo refrigerante); 4) salgadinhos industrializados vendidos em pacotes; 5) biscoito ou bolachas salgadas ou doces; e 6) balas, confeitos, doces e outros.

^b^ O escore de disponibilidade de estruturas e materiais para atividade física é composto pelos seguintes itens: quadra de esporte; pelo menos uma quadra de esporte coberta; pista de corrida/atletismo; pátio; piscina; vestiários; material esportivo ou para jogos e brincadeiras.

O escore Disponibilidade de estruturas e materiais teve consistência interna abaixo do aceitável (alfa = 0,60) e a correlação entre os itens variou de -0,011 a 0,798. Os valores máximos foram extremos nas duas dependências administrativas, sendo observadas escolas com todas as sete estruturas avaliadas (100 pontos). Ainda, foram identificadas escolas públicas com nenhuma estrutura disponível, enquanto as escolas privadas tinham no mínimo uma estrutura disponível (resultados não mostrados). A pontuação média das escolas privadas (63,3; IC95% 60,6;66,0) foi maior que a das escolas públicas (41,6; IC95% 40,3;42,8). O escore nas escolas públicas foi relacionado à região e à localização do município, enquanto para as escolas privadas apenas à região. Maior pontuação entre as escolas públicas foi verificada nas regiões Sudeste (significativamente maior que Norte, Nordeste e Centro-Oeste) e Sul (significativamente maior que Norte e Nordeste), e a região Centro-Oeste foi a que apresentou melhor ambiente entre as escolas privadas. Escolas públicas localizadas em capitais apresentaram maior pontuação quando comparadas àquelas localizadas fora de capitais (Tabela 5).

## DISCUSSÃO

Utilizando dados de escolas públicas e privadas, distribuídas pelas cinco macrorregiões brasileiras, em capitais e não capitais, observou-se, a partir dos indicadores-síntese utilizados, um ambiente mais favorável à alimentação saudável em escolas públicas, enquanto as escolas privadas apresentaram melhor ambiente para a prática de atividade física. No entanto, considerando que a alimentação saudável e a prática regular de atividade física contribuem para diminuição do risco de DCNT e que o ambiente escolar deve promover práticas saudáveis para prevenir essas doenças^24^, a despeito das diferenças encontradas, há que se melhorar o ambiente de escolas públicas e privadas brasileiras.

Em relação ao ambiente alimentar, existem diferenças significativas entre escolas públicas e privadas – sendo as mais evidentes oferta de alimentação escolar, presença de cozinha em condições de uso (o que permite o preparo de refeições) e presença de cantinas –, bem como entre os tipos de alimentos comercializados nas cantinas.

A existência do PNAE nas escolas públicas, que garante a oferta universal de alimentação aos estudantes da educação básica^5^, justifica os resultados encontrados. O PNAE é um importante elemento do ambiente alimentar escolar, visto que o consumo frequente da alimentação oferecida está relacionado a uma melhor qualidade da alimentação de estudantes brasileiros^14^. Ainda, dado que o PNAE se restringe às escolas públicas, isso pode contribuir para a elevada presença de cantinas nas escolas privadas^25^. Considerando os alimentos comercializados em cantinas, a baixa frequência desses estabelecimentos nas escolas públicas provavelmente contribuiu para a maior pontuação dessas escolas no escore que desenvolvemos.

Os resultados do presente trabalho são convergentes com a literatura sobre o tema. Dados do Estudo de Riscos Cardiovascular em Adolescentes (Erica), realizado com 1.247 escolas de 124 municípios, encontraram maior oferta de alimentos ultraprocessados nas escolas privadas, que mais frequentemente tinham estabelecimentos de venda de alimentos em seu interior e no entorno^8^. Outro estudo realizado na cidade de Santos (SP) avaliou o ambiente alimentar no entorno de escolas e observou que os estabelecimentos mais próximos das escolas ofertavam maior quantidade de alimentos ultraprocessados^26^.

Conhecer o ambiente alimentar das escolas brasileiras é importante uma vez que a literatura aponta que a presença de cantinas e máquinas de autosserviço no interior e no entorno, com grande oferta de alimentos ultraprocessados, exerce forte influência sobre a chance de consumo desses alimentos pelos estudantes^11^. Cabe também ressaltar que, a despeito da ausência de regulação nacional, existem leis e regulamentações estaduais e municipais que visam controlar e/ou proibir a oferta de alimentos não saudáveis nas escolas^4,7,15,27^. No entanto, a frequência de comercialização desses alimentos ainda é elevada.

Em relação ao ambiente para a prática de atividade física, as escolas da rede privada apresentaram mais estruturas/itens favoráveis em seu interior. Houve expressiva diferença nos valores em comparação com a rede pública, uma vez que piscina, pista de corrida e vestiário foram observados em frequência inferior (com diferenças de 32,9, 6,2 e 45,3 pontos percentuais, respectivamente) nestas. A presença do pátio e dos materiais esportivos foi observada na maioria das escolas brasileiras, e pode-se considerar que estes são espaços e itens fundamentais para o funcionamento das escolas e realização das aulas de educação física. Ainda assim, podem não ser o bastante para a promoção da atividade física, uma vez que maior quantidade de estruturas/itens disponíveis resulta em maior chance de nível mais elevado de atividade física entre os alunos^10^.

Evidências apontam a relação entre a presença de estruturas e os níveis de atividade física. Itens como campo de beisebol e academia coberta, bem como o tamanho da escola, apresentaram associação significativa com a prática de atividade física no Canadá^17^. Além disso, estudos realizados na Suécia, Bélgica e nos Estados Unidos verificaram que ambientes com melhores estruturas físicas podem aumentar significativamente os níveis de atividade física dos estudantes^28,29^.

Quanto à literatura nacional, estudo realizado na cidade de Londrina (PR) com 1.562 alunos da educação infantil de 20 escolas observou que pátio coberto era uma estrutura comum a todas as escolas, em contrapartida, nenhuma delas oferecia atividades físicas extracurriculares e aulas de educação física, e menos de um terço tinha salas de recreação, parques e brinquedos portáteis^30^. Análise de dados da PeNSE 2012, que contemplou 109.104 estudantes brasileiros de 2.842 escolas, mostraram que a maioria das escolas era pública, tinha pelo menos uma quadra poliesportiva e oferecia atividades esportivas extracurriculares (64%), e que a disponibilidade de um pátio escolar com atividade física dirigida pelo professor, piscina em condições de uso e vestiário em condições de uso foram menos comuns. Ainda, escolas com pelo menos uma estrutura/instalação para atividade física resultaram em maior chance de participação dos estudantes na educação física, entretanto, para aumentar a atividade física no lazer e o nível de atividade física total, pelo menos quatro e duas estruturas/instalações, respectivamente, eram necessárias. Além disso, as atividades esportivas extracurriculares nas escolas se associaram positivamente à atividade física no lazer e ao nível de atividade física^10^.

Os escores desenvolvidos neste estudo contribuem com a literatura de avaliação dos ambientes escolares alimentar e para a prática de atividade física, explorando algumas de suas dimensões a partir de variáveis coletadas em um grande inquérito nacional, realizado periodicamente.

A literatura considera que um alfa Cronbach acima de 0,70 é aceitável na avaliação de consistência interna de indicadores. No nosso estudo, o escore de Disponibilidade de alimentos e bebidas apresentou alfa de 0,79. Porém, foi de 0,60 para o escore de Disponibilidade de estruturas e materiais para a prática de atividade física, o que em parte pode ser justificado pelo limitado número de itens que compõem o indicador^22,23^. Além disso, indicadores-síntese para avaliação de ambiente são de complexa mensuração e não serão perfeitamente cobertos por quaisquer conjuntos de variáveis, fazendo com que valores até 0,60 sejam aceitáveis^31^. De qualquer forma, os indicadores devem ser interpretados com cautela, uma vez que todos os itens, independentemente de serem positivos ou negativos e de sua frequência, tiveram o mesmo peso. A despeito da importância da presença de estruturas de produção e oferta de alimentos e bebidas para uma avaliação ampliada do ambiente alimentar escolar, o escore sobre a disponibilidade de tais estruturas apresentou consistência muito baixa, provavelmente em função dos poucos itens disponíveis na PeNSE para compô-lo. Neste sentido, optamos por manter a descrição das variáveis isoladas, mas não apresentar os valores para o escore.

Escolas sem dados disponíveis em relação às variáveis de estudo foram excluídas, sendo essas 93 escolas das análises sobre ambiente alimentar e seis escolas das análises sobre o ambiente para a prática de atividade física. Análises de sensibilidade considerando os valores faltantes como ausência do item desejável foram realizadas, não tendo sido identificadas diferenças entre os resultados. Considerando o tamanho da amostra de escolas e as análises de sensibilidade realizadas, tais exclusões não impactaram os resultados.

É importante destacar alguns pontos fortes do presente estudo. A abordagem descritiva dos ambientes de interesse em amostra representativa de escolas das cinco macrorregiões, juntamente com os escores construídos a partir das perguntas do questionário da PeNSE, pode ser considerada inovadora para a avaliação desses ambientes. Considerando que a PeNSE acontece periodicamente, a avaliação dos escores, juntamente com a descrição de cada um dos itens que os compõem, pode contribuir para o monitoramento do ambiente escolar brasileiro, no entanto, há que se avançar na validação dos escores.

Nossos achados indicam que o ambiente escolar é um espaço de ampla e variada presença de elementos que podem contribuir para a alimentação e a prática de atividade física. Conclui-se que o ambiente alimentar de escolas públicas foi mais favorável à promoção da alimentação saudável que o de escolas privadas, sendo o oposto verificado para o ambiente para a prática de atividade física, ainda que importantes diferenças tenham sido observadas principalmente entre macrorregiões. Estes achados reforçam a importância do incentivo à adoção de práticas e políticas promotoras da saúde no ambiente escolar.
